# Influence of household demographic and socio-economic factors on household expenditure on tobacco in six New Independent States

**DOI:** 10.1186/1471-2458-7-222

**Published:** 2007-08-30

**Authors:** Mamuka Djibuti, George Gotsadze, George Mataradze, Akaki Zoidze

**Affiliations:** 1 Curatio International Foundation, Tbilisi, Georgia; 2 Department of Public Health, Tbilisi State Medical University, Georgia

## Abstract

**Background:**

To identify demographic and socio-economic factors that are associated with household expenditure on tobacco in Azerbaijan, Georgia, Kazakhstan, Kyrgyzstan, Russian Federation, and Tajikistan.

**Methods:**

Secondary analysis of the data available through the World Bank Living Standards Monitoring Survey conducted in aforementioned countries in 1995–2000. The role of different variables (e.g. mean age of household members, household area of residence, household size, share of adult males, share of members with high education) in determining household expenditure on tobacco (defined as tobacco expenditure share out of total monthly HH consumption) was assessed by using multiple regression analysis.

**Results:**

Significant differences were found between mean expenditure on tobacco between rich and poor – in absolute terms the rich spend significantly more compared with the poor. Poor households devote significantly higher shares of their monthly HH consumption for tobacco products. Shares of adult males were significantly associated with the share of household consumption devoted for tobacco. There was a significant negative association between shares of persons with tertiary education within the HH and shares of monthly household consumption devoted for tobacco products. The correlation between household expenditures on tobacco and alcohol was found to be positive, rather weak, but statistically significant.

**Conclusion:**

Given the high levels of poverty and high rates of smoking in the New Independent States, these findings have important policy implications. They indicate that the impact and opportunity costs of smoking on household finances are more significant for the poor than for the rich. Any reductions in smoking prevalence within poor households could have a positive economic impact.

## Background

In the New Independent States (NIS), there has been a marked socio-economic and structural change, which followed the end of the socialist regime in 1989 and 1992, and the subsequent attempt of transition to a market economy. This period was characterized by steady increase in poverty in all transition countries – with dramatic increase in some. Countries like, Azerbaijan, Georgia, Kyrgyz Republic and Tajikistan are now among the poorest in the region [1].

Smoking may have serious implications for poverty. The opportunity cost of money spent on cigarettes is obviously higher for people living on low incomes – saving money spent on tobacco products could help feed families [2]. Data from various countries clearly show that tobacco is often a significant part of family expenditures [3-6].

According to the results of recent studies, smoking rates among men in the Former Soviet Union are very high and that female rates, while yet low, appear to be increasing [7-10]. Namely, smoking rates in men in the six NIS countries of interest vary from 50% to 65% and rates in women from 2% to 16% [11].

The aim of the proposed study was to assess the economic burden of smoking at a HH level in six NIS countries (Azerbaijan, Georgia, Kazakhstan, Kyrgyzstan, Russian Federation, and Tajikistan), using *Living Standard Monitoring Survey (LSMS) *data sets and thereby to provide information critical to the development of a tobacco control policy.

## Methods

### Design and data sources

The basic design of the study has been secondary analysis of the data available through World Bank LSMS. LSMS data files and necessary supporting documentation (survey instruments and questionnaires) were obtained for all six countries. The year of LSMS and HH count included into the survey are as follows: Azerbaijan – 1995/2,000; Georgia – 1999/3,300; Kazakhstan 1996/2,000; Kyrgyzstan – 1998/3,000; Russian Federation – 2000/4,000; Tajikistan – 1999/2,000.

In each of these six countries, LSMS collected data on household demographic, economic and social characteristics, namely a) number, age, and sex of HH members; b) education level of HH members; c) employment status of HH members; d) HH monthly expenditure on tobacco; e) HH monthly expenditure on alcohol; f) HH monthly expenditure on food; and g) HH total monthly expenditures.

All data on expenditures were collected at HH (and not at individual) level, which made us decide to use HH as the unit of analysis. Other socio-demographic data were collected at the individual level. Thus to be able to conduct HH level analysis, it was necessary to construct composite variables – socio-demographic indexes, details on which are given in the text below.

### Variables

To investigate differences in HH expenditure on tobacco between rich and poor households, we have developed a composite measure, the HH Poverty index. HHs in each country were divided into 5 equal groups based on their monthly total per capita expenditure (population expenditure quintiles). Households belonging to the lowest expenditure quintile were classified as "poor" and households belonging to the highest expenditure quintile were classified as "rich".

It should be said that the measurement of per capita expenditures rules out the possibility to attach different economies of scale to households of different types [12]. Economies of scale arise in many ways – for example, by sharing certain expenditures such as expenditures on housing, utilities, cars, etc. Apart from household size, the age or gender of household members may also influence the amount of consumption, for example food. Other judgments may be used, as a result a wide variety of equivalence scales are used in various countries, and there is no accepted equivalence scale in the FSU [13-16]. Hence, we used per capita expenditure measure – by far the most widespread rule applied in practice, which assumes that all family members receive the same fraction of household expenditures [17]. Before deciding to proceed with this approach, however, we conducted sensitivity tests by using different scales according to the methodology adopted by The World Bank [18], which revealed no significant variations in the household mean expenditures on tobacco by expenditure quintile groups.

To investigate variations in HH expenditure on tobacco by HH structure, i.e. number, age, gender, employment, and education level of HH members, we have defined the following composite variables – socio-demographic indexes: a) HH Size Index – number of HH members; b) HH Adult Index – share of 15 to 65 year old people in a HH; c) HH Elderly Index – share of people age more than 65 years in a HH; d) HH Children Index – share of children under 15 years of age in a HH; e) HH Adult Female Index – share of adults 15 to 65 year old females in a HH; f) HH Adult Male Index – share of adults 15 to 65 year old males in a HH; g) HH Gender Index – HH adults 15 to 65 year old female/male ratio; h) HH Employment Index – share of HH members in permanent employment; and f) HH Education Index – share of HH members with high (university) education.

### Statistical analysis

Expenditure on tobacco (both mean and total) was calculated for each expenditure quintile group separately. For comparison of the mean expenditure on tobacco between groups we used analysis of variance (ANOVA). For comparison of urban vs. rural differences in the mean expenditures on tobacco by expenditure groups we used Student's t test.

Tobacco expenditure was also calculated as a proportion of HH total monthly spending for each household and the mean percentage in each expenditure group was then calculated. The mean expenditure share (%) between expenditure groups was then compared using analysis of variance (ANOVA). Similar analysis between different expenditure groups was done for urban and rural HHs separately. For evaluating urban versus rural differences by expenditure groups we used Student's t test.

Association between HH expenditure on tobacco and alcohol was explored by estimating Pearson's correlation coefficient between HH expenditure on alcohol and tobacco as absolute expenditures. Azerbaijan was excluded from this analysis given that there was no separate data on HH expenditure on tobacco and alcohol.

Association between HH socio-demographic indexes and HH expenditure on tobacco as proportion of total monthly HH consumption was explored by using univariate linear regression analysis. In addition to aforementioned indexes, in the models of each country we included the variables such as HH per capita total monthly spending and HH area of residence.

In order to define the socio-demographic factors/indexes that are independently associated with high share of HH's expenditure on tobacco, we constructed multiple linear regression models. In the model for all countries, we entered the variables that proved to be significant in the univariate analysis. We did not include those variables that strongly correlated with each other to avoid the effect of colinearity. In order to reduce the chance of type I error due to a high number of significance testing, all p values were set to <0.01.

## Results

### Variation in HH expenditure on tobacco by HH economic index

In all six countries significant differences were found between mean expenditure on tobacco between rich and poor – namely, the rich spend significantly more compared with poor (Table 1).

**Table 1:** Mean expenditure on tobacco by HHs per month (in national currency)

**Expenditure group**	**Azerbaijan (AZM)**	**Georgia (GEL)**	**Kazakhstan (KZT)**	**Kyrgyzstan (KGS)**	**Russia (RUB)**	**Tajikistan (TJS)**
**Poor**	4,510.0	1.9	73.1	2.7	18.5	254.4
**2**	10,676.7	7.7	125.0	4.8	35.5	347.5
**3**	16,609.1	15.0	190.1	6.1	57.2	491.5
**4**	21,904.7	27.7	242.5	9.1	89.0	683.8
**Rich**	48,366.7	57.0	361.8	13.3	141.6	1378.3
**Total**	20413	**22.4**	**198.5**	**7.2**	68.4	**631.0**
**F**	**48.8**	**100.2**	**44.39**	**51.96**	**117.2**	**17.53**
**Sig**.	**0.000**	**0.000**	**0.000**	**0.000**	**0.000**	**0.000**

GEL, Georgian Lari; KZT, Kazakhstan Tenge; TJS, Tajikistan Somoni; KGS, Kyrgyzstan Som; AZM, Azerbaijan Manat; RUB, Russian Rouble.

As for urban vs. rural differences in the mean expenditures on tobacco by expenditure groups, it was found that among the poor, those living in rural areas had higher expenditures compared with those living in urban places, and this was true for all countries except Azerbaijan. The opposite trend was found among the rich, namely, those living in urban areas had higher expenditures on tobacco. It should be said, however, that these differences were statistically significant for Georgia and Kyrgyzstan only. Russia LSMS did not provide data on urban/rural affiliation of HHs. Therefore, we could not assess urban vs. rural differences in HH expenditures on tobacco.

As can be seen from Table 2, poor HHs have significantly higher expenditures on tobacco as proportion of total monthly HH outgoings in all countries excluding Georgia and Azerbaijan.

**Table 2:** Expenditure on tobacco as proportion (%) of HH's total monthly spending by countries

**Expenditure group**	**Azerbaijan**	**Georgia**	**Kazakhstan**	**Kyrgyzstan**	**Russia**	**Tajikistan**
**Poor**	3.28	1.54	2.47	0.53	2.75	1.32
**2**	3.43	1.89	1.67	0.49	2.08	0.79
**3**	3.67	1.99	1.63	0.43	2.06	0.78
**4**	3.31	2.01	1.41	0.42	2	0.77
**Rich**	3.66	1.79	1.19	0.31	1.4	0.78
**Total**	**3.47**	**1.85**	**1.67**	**0.43**	**2.06**	**0.89**
**F**	**0.39**	**1.092**	**6.44**	**5.19**	**10.59**	**3.34**
**Sig**	**0.82**	**0.359**	**0.000**	**0.000**	**0.000**	**0.01**

When we did separate analysis to compare tobacco expenditure shares between urban and rural HHs by expenditure quintile groups, it was found that rich HHs living in urban areas spend significantly higher portion of their monthly total spending on tobacco compared with rich HHs residing in rural areas (the difference was statistically significant for Georgia, Kazakhstan, and Kyrgyzstan). Differences between urban and rural HHs belonging to the poorest groups was not significant in any of these countries. Once again this analysis could not be conducted for Russia as no data on urban/rural status was provided.

### The association between HH expenditure on tobacco and alcohol

The correlation between absolute expenditures on tobacco and alcohol was found to be positive, rather weak, but statistically significant in all five countries (Pearson's r being 0.145, 0.201, 0.106, 0.204, and 0.304 for Georgia, Kazakhstan, Kyrgyzstan, Russia, and Tajikistan, respectively; p < 0.01 for all countries) indicating that HHs that spend more on tobacco are also likely to spend more on alcohol.

#### The factors independently associated with high share of HH's expenditure on tobacco

All variables (per capita monthly total spending, HH area of residence, mean age of HH members plus all socio-demographic indexes as described above) were included in univariate linear regression analysis for each country. Variables that proved to be significantly associated with the dependent variables (tobacco expenditures share on total monthly spending) were then entered in multiple linear regression models. Table 3 presents only those variables that were significantly associated with the dependent variable in five countries. In Azerbaijan, none of the variables had significant association with the dependent variable (tobacco & alcohol expenditure share on total monthly spending).

**Table 3:** Results from Multiple Linear Regressions for 5 countries – Dependent Variable: tobacco expenditure share out of total monthly spending

**Country/Independent Variable**	**Regression coefficient (95% CI)**	**p Value**	**Adjusted R**^2^
**Georgia**			
Mean age of HH members	-0.020 (-0.032 to 0.008)	0.001	0.039
Adult females (15 to 65) share out of total number of HH members	0.024 (0.016 to 0.031)	0.000	
Adult males (15 to 65) share out of total number of HH members	-0.012 (-0.019 to -0/005)	0.001	
**Kazakhstan**			
HH per capita total monthly spending	-0.000 (0.000 to 0.000)	0.000	0.045
Adult males (15 to 65) share out of total number of HH members	0.029 (0.02 to 0.036)	0.000	
**Kyrgyzstan**			
Adult females (15 to 65) share out of total number of HH members	-0.003 (-0.004 to 0.001)	0.003	0.037
Adult males (15 to 65) share out of total number of HH members	0.007 (0.005 to 0.009)	0.000	
Share of employed plus self employed out of total HH members	0.002 (0.001 to 0.003)	0.003	
Share of persons with high education out of total HH members	-0.003 (-0/004 to -0.001)	0.004	
**Tajikistan**			
Adult males (15 to 65) share out of total number of HH members	0.015 (0.006 to 0.022)	0.000	0.015
**Russia**			
Mean age of HH members	-0.021 (-0.027 to -0.012)	0.000	0.06
HH per capita total monthly spending	-0.000 (0.000 to 0.000)	0.000	
Adult males (15 to 65) share out of total number of HH members	0.032 (0.026 to 0.038)	0.000	
Share of persons with high education out of total HH members	-0.010 (-0/015 to -0/005)	0.000	

Adult male's share was significantly associated with tobacco expenditure share in all countries (except Georgia, where adult female's share emerged as independent positive predictors). Urban area of residence emerged as independent predictor for higher expenditure share on tobacco in Kyrgyzstan only. Share of employed persons in a HH was independently associated with higher expenditure share on tobacco only in Kazakhstan.

It should be stressed that all models explained from 1.5 to 6% of the variance of the tobacco expenditure share, which indicates the limitation of these constructed models.

## Discussion

A major limitation of the study has been that Azerbaijan LSMS collected data on HH expenditures on tobacco aggregated with expenditures on alcohol, and there was no way to disaggregate these data. Therefore, results of the analysis of Azerbaijan data should be interpreted with some caution. In Georgia, LSMS data was obtained from the local State Department of Statistics (and not from the World Bank as was done for other countries), which raises concern with regard to data validity considering that the data set has not probably gone through careful quality checking procedures.

Results of this analysis clearly indicate that tobacco use poses greater economic burden on poor HHs in Russia, Kazakhstan, Tajikistan, and Kyrgyzstan where tobacco expenditure share on HH's total monthly spending among poor is 2.75%, 2.47%, 1.32%, and 0.53%, respectively. Further research is needed to disclose the factors determining these differences in various countries. The opposite findings for Georgia and Azerbaijan confirm the authors concern with regard to the validity of Georgian LSMS data and the above statement that the data for Azerbaijan should be interpreted with some caution, since expenditures on tobacco and alcohol are pooled together.

Situation in regard with HH tobacco expenditures share out of the total expenditure seems to be comparable or slightly better in NIS compared with other countries: in the Bangladesh survey, on average for poor HHs, 2.46% of total expenditures were allocated for tobacco products [5]. In Sri Lanka, the poorest households spent an average of just over 3% of total expenditures on tobacco products [4]. In Bulgaria, the low and lower-middle income group spent about 4.9% of total income on cigarettes [6]. Nevertheless, tobacco expenditure share is still high and may exacerbate the effects of poverty and cause significant deterioration in living standards among the poor.

The fact that adult males share was significantly associated with tobacco expenditure is in line with the results of studies evaluating patterns of smoking in the former Soviet Union [7-9,11,19], where smoking was more common among males of all ages and areas.

There was significant negative association between share of persons with university education and tobacco expenditure share in Kazakhstan, Kyrgyzstan, and Russia. This is generally consistent with recent survey findings, which show a link between smoking and lack of education in eight countries of the former Soviet Union [20].

Urban area of residence emerged as independent predictor for higher expenditure share on tobacco in Kyrgyzstan. There was no other country showing similar association. This may reflect the findings of other studies focusing on the same region and showing that smoking among men varies relatively little by area of residence [9,19,20].

Although there have been studies showing that smoking rates are increased by material hardship [20], particularly unemployment [7,8], our study failed to identify an independent association between unemployment and tobacco expenditure share out of HH total monthly spending in all countries but Kyrgyzstan. This may be determined by relative limited contribution of formal employment to HH income in these countries [21].

## Conclusion

In absolute terms rich households tend to spend more on tobacco than poor households. However, poor HHs spend a significantly higher portion of their household budget on tobacco compared with rich HHs. HHs with more males have significantly higher expenditure share on tobacco. HHs with more educated persons have significantly lower expenditure share on tobacco.

## Competing interests

The author(s) declare that they have no competing interests.

## Authors' contributions

MD and GG conceived the research and study design, lead the analysis and drafted the manuscript. GM and AZ assisted with drafting of the manuscript. All authors read and approved the final manuscript.

## Pre-publication history

The pre-publication history for this paper can be accessed here:


